# A systematic review of the impact of climate-related displacements on food and water security in Mozambique and Zimbabwe

**DOI:** 10.4102/jamba.v18i1.1965

**Published:** 2026-02-28

**Authors:** Sindiso Ndlovu, Gracsious Maviza

**Affiliations:** 1 International Centre for Tropical Agriculture (CIAT), Pretoria, South Africa; 2 Department of Demography and Population Studies, School of Public Health and Social Sciences, University of the Witwatersrand, Johannesburg, South Africa

**Keywords:** climate-induced displacements, water security, food security, droughts, cyclones, livelihoods, human security, Mozambique, Zimbabwe

## Abstract

Climate-related displacements in Southern Africa have escalated because of an increasing frequency and severity of extreme weather events, particularly cyclones, floods and droughts. Mozambique and Zimbabwe are among the most affected countries, where repeated climate shocks have displaced communities and disrupted critical livelihood systems. However, evidence on how these displacement processes specifically influence food and water security remains insufficiently synthesised. This study examines this gap through a systematic review guided by the question: How do climate-related displacement events affect food and water security among populations in Mozambique and Zimbabwe? Following the PRISMA framework, 32 peer-reviewed studies published between 2010 and 2024 were identified using PICO-derived search terms. Studies were included if they explicitly examined displacement and its implications for food and water security. The Mixed Methods Appraisal Tool (MMAT) was used to assess methodological quality, and a Most Similar Systems Design (MSSD) approach enabled comparison of governance responses shaping outcomes for displaced populations. Findings indicate that climate-related displacement intensifies socio-economic vulnerabilities, reduces access to land and safe water, and heightens resource competition in receiving areas. These pressures undermine household food availability, dietary diversity and water reliability, while increasing exposure to protection risks. Weak institutional capacity and inconsistent policy frameworks further constrain sustainable recovery. This review advances knowledge by synthesising dispersed evidence and identifying critical gaps in governance, planning and service provision.

**Contribution:**

It emphasises the need for integrated, human-security-oriented interventions that strengthen resilience, safeguard essential resources and ensure displaced populations are effectively included in national adaptation and disaster-risk-reduction strategies.

## Introduction

Climate change presents an escalating global challenge with profound implications for societies, economies and ecosystems. Its effects on humanity have been catastrophic and detrimental, undermining all facets of human security. Southern Africa is especially vulnerable to the negative outcomes related to climate change (Jacobs & Almeida 2020; Maviza et al. 2024; Mugabe et al. 2021). Mozambique and Zimbabwe continue to experience recurring cyclones, floods and droughts that displace communities and disrupt critical livelihood systems. These effects are further compounded by the socio-economic and environmental fragilities experienced in the region (Baez, Caruso & Niu 2020; Mugabe et al. 2021; Ndlovu et al. 2024b). Although climate-induced mobility affects several dimensions of human security, this review focuses specifically on food and water security, which are the most immediate and consistently documented impacts of displacement. These insecurities commonly arise from the loss of land, damaged infrastructure and reduced access to stable sources of food and safe water. Countries such as Mozambique, Malawi and Zimbabwe are uniquely affected by extreme weather events, including cyclones, floods and prolonged droughts, which have ‘sudden hurtful disruptions in the patterns of daily life’ (Baez et al. 2020; Mavhura 2020; Nhamo, Dube & Saurombe 2021; Spiegel, Kachena & Gudhlanga 2023). Notably, livelihoods for most communities in these countries are agro-based and heavily dependent on seasonal rainfall, leaving rural communities particularly susceptible to climate shocks, threatening food and water security and intensifying human insecurity.

In efforts to adapt to the adverse effects of climate change, there has been a notable trend and increase in rural-to-urban migration, further complicating this dynamic (Ndlovu et al. 2024a; Nzima & Maviza 2023; Spiegel et al. 2023; Trummer et al. 2023). Displaced populations frequently move to urban centres seeking improved living conditions, yet most African cities are ill-equipped to absorb this influx (Kurete 2019; Spiegel et al. 2023). This has often led to the sprouting of overcrowded informal settlements in these urban areas, which have increasingly become prone to land conflicts, health crises and environmental degradation, as evidenced by cholera outbreaks following Cyclone Idai (Aderinto 2023; Spiegel et al. 2023). This migration-driven urbanisation, fuelled by environmental stress and socio-economic hardship, exacerbates human insecurity and poses long-term threats to regional stability. The literature on climate change, displacement and human security reveals both areas of agreement and important nuances regarding how food and water insecurity emerge in contexts affected by environmental stress. A substantial body of research demonstrates that climate variability and extreme events such as droughts and floods adversely affect agricultural productivity and water availability, leading directly to food and water insecurity for vulnerable households (FAO 2018; Muzerengi, Gandidzanwa & Chirubvu 2023). For example, prolonged drought and shifting rainfall patterns reduce crop yields and limit water resources necessary for irrigation and daily use, undermining both food production and access to potable water in rural Africa (FAO 2018; Muzerengi et al. 2023). These disruptions often intersect with wider livelihood vulnerabilities and can precipitate migration or displacement as affected populations seek more secure conditions. At the same time, some studies emphasise that the relationship between climate impacts, displacement and resource insecurity is mediated by structural and socio-institutional factors rather than being a simple causal pathway. Governance limitations, weak land-use planning and inequitable water management can magnify the effects of climatic stressors on food and water security independent of displacement itself (Yeboah 2024; Morales-Muñoz et al. 2020). Research also shows that where adaptive capacity is limited, such as in settings with poor infrastructure and limited social protection, the compounded effects of climate hazards and socio-economic fragilities produce more severe food and water insecurity (Yeboah 2024). Recognising these divergent perspectives strengthens the analytical grounding of this review by highlighting that climate-related displacement interacts with existing vulnerabilities, governance contexts and adaptation capacities to shape the severity and persistence of food and water insecurity. This systematic literature review examines the implications of climate-induced displacements on food and water security in Mozambique and Zimbabwe, focusing on the interconnected issues of food, land and water security-related tensions. Mozambique and Zimbabwe offer important comparative insights because both countries face recurrent climate hazards but apply different approaches to disaster management, resettlement and land administration. These contextual differences influence how displaced populations experience food and water insecurity and shape the effectiveness of state and non-state responses. Against this background, the objectives of this review are as follows: to examine how climate-induced displacement affects food and water security in Mozambique and Zimbabwe; to identify the key food and water security risks and adaptation measures presented in the literature; and to assess the effectiveness of government and international responses in addressing these risks. Through this synthesis, the review aims to clarify areas of agreement and disagreement in existing research and to highlight the contextual and governance-related factors that shape human security outcomes for displaced populations. By examining these specific dimensions, the study highlights how displacement affects the stability, livelihoods and overall resilience of affected populations, emphasising the urgent need for targeted policy interventions that enhance adaptive capacities and mitigate conflict risks in these regions. The choice of these two countries for this study is informed by their exposure to climate-induced hazards, high levels of poverty and the significance of agriculture in rural livelihoods. These factors make them critical areas for examining the multifaceted effects of climate-induced displacement on human security.

### Climate change, displacement and human security in Zimbabwe and Mozambique: An overview

Climate change is increasingly recognised as a key driver of displacement and a threat to human security, particularly in vulnerable regions such as Southern Africa (Jacobs & Almeida 2020; Mutizwa 2023; Ndlovu et al. 2024a, 2024b; Rossi et al. 2024; Spiegel et al. 2023). Climate-related displacement represents a critical area of concern as the impacts of climate change intensify (Maviza et al. 2024; Nzima & Maviza 2023; Spiegel et al. 2023). Southern Africa’s climate is increasingly characterised by more frequent and intense cyclones and prolonged droughts, each contributing to distinct displacement patterns (Chatiza 2019; Mugabe et al. 2021; Trummer et al. 2023). Climate-driven events such as floods, cyclones and droughts continue to displace communities, often amplifying existing socio-economic vulnerabilities (Mucherera & Spiegel 2022; Mugambiwa & Makhubele 2021; Neumann et al. 2013; Rossi et al. 2024). Zimbabwe and Mozambique have fragile economies and are highly exposed to climate-related hazards, which have significantly impacted their populations, through displacement and heightened insecurity (Jacobs & Almeida 2020; Chatiza 2019; Mugabe et al. 2021; Mutizwa 2023). Both countries experience recurrent extreme weather events and socio-economic constraints, creating a high level of susceptibility to climate-induced displacements. Mozambique, in particular, is the most affected, reflecting its higher exposure and vulnerability to severe climatic events (Ndlovu et al. 2024b; Spiegel et al. 2023). In Mozambique, cyclones and tropical storms such as Idai and Kenneth have resulted in widespread internal displacement, forcing communities to relocate as infrastructure and homes were destroyed (Jacobs & Almeida 2020; Mugabe et al. 2021; Neumann et al. 2013; Rossi et al. 2024). Recent cyclones and tropical storms, including Idai (2019) and Kenneth (2019), displaced over 400 000 people, inflicting extensive damage to infrastructure and housing. More recently, Cyclone Freddy (2023) displaced approximately 184 000 individuals, further exacerbating vulnerabilities and forcing communities to relocate because of severe flooding and infrastructural damage. These events have created cycles of displacement and reconstruction that strain national resources and local communities (Tirivangasi et al. 2023). Similarly, Zimbabwe has faced the devastating impacts of these cyclones and floods, particularly in the eastern regions bordering Mozambique (Chatiza 2019; Mavhura 2020; Mugambiwa & Makhubele 2021; Mutizwa 2023; Nhamo et al. 2021). Additionally, one of the country’s unique drivers of displacement has been prolonged droughts beyond the floods and cyclones. These climate-related challenges threaten agricultural livelihoods in both countries, resulting in large-scale displacement and internal and cross-border mobility. Economic fragility compounds the effects of climate change in both nations, prompting many to migrate irregularly to neighbouring countries in search of improved living conditions (Ndlovu et al. 2024a, 2024b; Nzima & Maviza 2023). In Mozambique, displacement caused by natural hazards has led to the rise of overcrowded camps and resource scarcity (Jacobs & Almeida 2020; Baez et al. 2020; Ndlovu et al. 2024b). These dynamics further strain relations within communities and heighten tensions as displaced populations or migrants compete with locals who also live in precarious conditions (Maviza et al. 2024; Mugabe et al. 2021; Mutizwa 2023). As this happens, it significantly impacts human security in the two countries (Mutizwa 2023).

The concept of human security broadens the traditional focus on state security to encompass essential aspects of individual well-being, including food and water security, health and livelihoods (Crush 2013; Ekblom 2012; Maviza et al. 2024; Mutizwa 2023). When climate-related events displace people, human security is often compromised, leading to complex challenges beyond the immediate physical risks of displacement (Mugabe et al. 2021; Muromo & Mashingaidze 2024; Spiegel et al. 2023; Trummer et al. 2023). As such, human security in both countries is deeply impacted by climate-induced displacement, with food, water and land insecurity and inadequate access to healthcare, among others, as common challenges. In these contexts, women and children from displaced populations are disproportionately affected, facing heightened risks of gender-based violence and limited access to social services (Diab & Scissa 2023; Ndlovu et al. 2024a; Nzima & Maviza 2023). In Zimbabwe and Mozambique, disruptions from climate-induced displacements place immense strain on food and water resources, livelihoods and social stability, heightening the vulnerability of affected populations. In response to these growing climate-related challenges that threaten human security in both countries, governments have devised several policies and response strategies to address the urgent situation. For example, in Mozambique, the government developed disaster risk reduction (DRR) frameworks but requires more investment in resilience-building (Spiegel et al. 2023), while Zimbabwe’s National Climate Policy emphasises adaptation but faces implementation challenges (Chatiza 2019; Mavhura 2020). The two countries have experienced environmental stressors that are diverse yet interrelated, underscoring the urgent need for adaptive strategies tailored to these distinct challenges (Tirivangasi et al. 2023).

## Research methods and design

**FIGURE 1:**
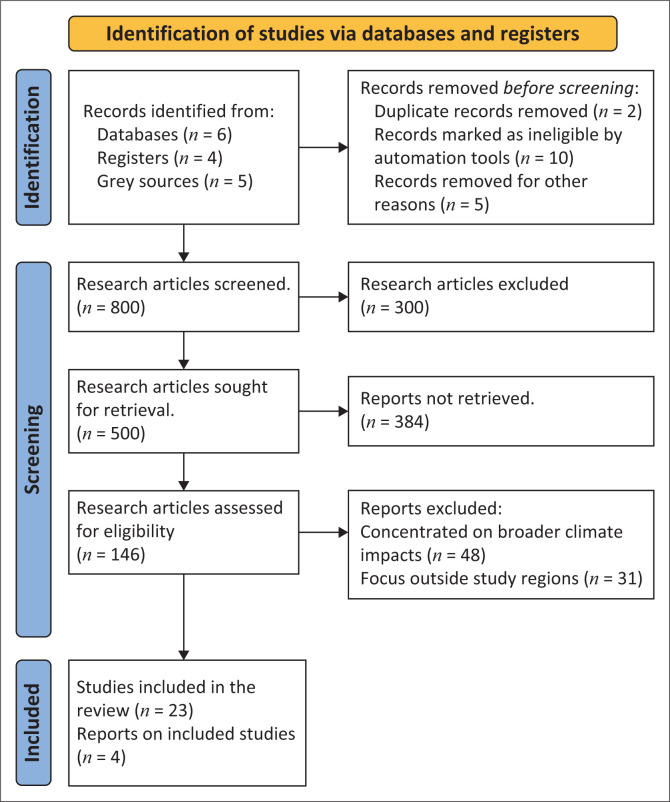
Flow diagram for systematic review search for literature.

This study employed a systematic literature review to analyse food and water security threats posed by climate-induced displacement in Mozambique and Zimbabwe. The methodology was guided by the Preferred Reporting Items for Systematic Reviews and Meta-Analyses (PRISMA) 2020 framework (Page et al. 2021) to ensure transparency, replicability and comprehensive reporting. The search and selection process is detailed in the PRISMA flow diagram (Figure 1), and the corresponding checklist was adhered to throughout. The review was designed to identify, appraise and synthesise existing evidence on how displacement driven by climatic events undermines human security, with a specific focus on food and water security. It was structured to address the following research questions: (1) How does climate-induced displacement impact food and water security in Mozambique and Zimbabwe? (2) What are the associated food and water security risks and adaptation measures? (3) How effective are government and international response mechanisms in mitigating these risks? The review followed the PRISMA framework’s four stages: identification, screening, eligibility and inclusion. In the identification phase, searches were conducted across several academic and specialised databases: Scopus, Web of Science, JSTOR and PubMed (for health-related impacts). Databases focused on African literature, such as African Journals Online (AJOL), and grey literature sources like Google Scholar and United Nations (UN) agency repositories, including the United Nations High Commissioner for Refugees (UNHCR), the Food and Agriculture Organization (FAO), and the United Nations Children’s Fund (UNICEF), were also used to ensure inclusivity and relevance. Search terms included ‘climate-induced displacement’, ‘food security’, ‘water security’ and ‘human security’ ‘nutrition’ ‘health’ paired with geographic terms (‘Mozambique’ and ‘Zimbabwe’) to narrow the scope to pertinent case studies and themes. These searches, limited to English-language studies published from 2010 to 2024, initially yielded approximately 800 articles and reports. Duplicates and non-relevant studies were removed, reducing the pool to 500. Titles and abstracts were screened for relevance to displacement, food and water security and human security risks, yielding a subset of 146 closely aligned studies. Inclusion criteria focused on literature explicitly addressing climate-induced displacement in Mozambique and Zimbabwe, especially studies with empirical data on food and water insecurity, human security risks and policy responses. Studies concentrating on broader climate impacts without displacement-specific data were excluded, as were those focusing outside the study regions unless comparative insights were provided. Ultimately, 32 articles and reports met the inclusion criteria for detailed analysis, outlined in Figure 1, which highlights the flow diagram for the systematic review search. The study incorporated two CGIAR FOCUS Climate Security empirical reports from Zimbabwe and Mozambique applying the Climate Security and Pathway Analysis (CSPA) framework, along with grey literature from the International Organization for Migration (IOM), World Food Programme (WFP), FAO and the World Bank. Mozambique and Zimbabwe were selected as case studies because of their shared transboundary risks that arise from interconnected environmental, economic, social or political factors, making them difficult to manage within a single country. Both countries therefore face vulnerability to extreme weather events, including cyclones, floods and droughts, which escalate displacement and intensify human security threats. While other Southern African nations face similar challenges, these two countries provided a valuable comparative context, highlighting the interactions between displacement, socio-economic vulnerabilities and governance factors that shape distinct human security outcomes.

### Case study rationale

Mozambique and Zimbabwe were selected as comparative case studies because of their shared vulnerability to transboundary climate risks such as cyclones and droughts, yet distinct socio-political and governance contexts. Both countries were selected as comparative cases following the Most Similar Systems Design (MSSD) logic, which examines similar contexts with differing outcomes to isolate causal factors (Seawright & Gerring 2008). Both countries face escalating climate-induced displacement that intensifies human security threats, providing a valuable lens to examine the interaction between environmental shocks, displacement and security outcomes. Their comparability aligns with the MSSD, as explained in the analysis section. Data analysis and synthesis: A comparative analytical approach was employed using the MSSD (Landman 2008). This framework facilitated a structured comparison of Mozambique and Zimbabwe, treating them as similar cases (shared vulnerabilities) to isolate how differing independent variables, such as the type of primary climate hazard (e.g. coastal cyclones vs. inland droughts) and governance responses, influenced the dependent variables of displacement patterns and food or water security outcomes. To ensure methodological rigour and minimise bias, the quality of all 32 included studies was appraised using the Mixed Methods Appraisal Tool (MMAT), version 2018 (Hong, Gonzalez-Reyes & Pluye 2018). Two independent reviewers assessed each study, and any discrepancies were resolved through discussion and consensus to ensure objectivity. Data extraction was conducted using a standardised data extraction form developed specifically for this review. The form was structured around the PICO (Population, Intervention, Comparison, Outcome) framework to ensure consistency and comprehensiveness in capturing key study details (Methley et al. 2014), defining *Population* as displaced populations and host communities in Mozambique and Zimbabwe, *Intervention/Exposure* as climate-induced displacement events (e.g. cyclones, floods, droughts), *Comparison* as differing socio-political and environmental contexts within and between the two countries, and *Outcomes* as the measured effects on food security, water security, health, livelihoods and conflict.

### Ethical considerations

This article followed all ethical standards for research without direct contact with human or animal subjects.

## Results and discussion

### Impacts of climate-induced displacement on food and water security

This section discusses the complex pathways through which climate-induced displacement disrupts traditional food production systems and access to water in Mozambique and Zimbabwe. The analysis reveals a consensus in the literature on the severe impacts of these disruptions, while also noting critical divergences in the specific vulnerabilities and adaptive capacities observed in the two national contexts.

### Climate-induced displacement and food (in)security

The findings consistently highlight that climate-related displacement and food insecurity are intricately intertwined in Mozambique and Zimbabwe (Mugambiwa & Makhubele 2021; Mutizwa 2023). Extreme weather events such as cyclones, droughts and floods routinely disrupt agricultural production, deplete food reserves and exacerbate hunger, leading to a precarious existence for food-insecure communities, displaced and host communities alike (Mugambiwa & Makhubele 2021; Muromo & Mashingaidze 2024; Mutizwa 2023; Ndlovu et al. 2024b). In both countries, subsistence farmers, already dependent on fragile agricultural systems, face heightened vulnerabilities because of displacements triggered by these climate events, undermining local livelihoods and overall survival prospects (Jacobs & Almeida 2020; Chatiza 2019). The susceptibility of these regions is largely because of their reliance on rain-fed agriculture, leaving crops exposed to erratic climate patterns that result in severe losses (Cau & Agadjanian n.d.; Mugambiwa & Makhubele 2021; Ndlovu et al. 2024a).

The scale of impact is severe and well documented. Zimbabwe experienced its most severe drought in 40 years during the 2018/19 agricultural season. Over 5.5 million smallholder farmers faced near-total crop failure, leaving more than 50% of the population needing food assistance (Ndlovu et al. 2024a). Subsequent erratic rainfall, such as during the 2020–2021 season in Masvingo and Matabeleland, led to a 34% reduction in maize production, endangering the food security of millions (Mutizwa 2023). Similarly, in Mozambique, frequent droughts strain critical river basins like the Limpopo and Save, impacting approximately 3.3 million people (Ndlovu et al. 2024b). Nearly 1.9 million people in Mozambique, including approximately 40 000 in emergency situations, face high levels of acute food insecurity, with 71% concentrated in Cabo Delgado, Niassa, Nampula and Zambézia provinces (Ndlovu et al. 2024b). Cyclone events compound these chronic stresses with acute devastation. Cyclones Idai and Kenneth (2019) destroyed approximately 715 000 hectares of crops in Mozambique’s Sofala and Manica provinces on the eve of harvest, triggering widespread food shortages (Ekblom 2012; Mavhura 2020; Mugabe et al. 2021; Spiegel et al. 2023). In Zimbabwe, similar impacts were observed in Chimanimani, Chipinge and Tsholotsho districts, where cropland destruction worsened food insecurity among populations already displaced and dependent on subsistence farming (Chatiza 2019; Mutizwa 2023). A critical insight from the literature is that displacement itself becomes a multiplier of food insecurity. Displaced farmers often lose access to their land, seeds and livestock, crippling their capacity to resume production (Jacobs & Almeida 2020). The degradation of soil through flood-induced erosion has further reduced arable land quality, thereby intensifying food scarcity (Nhamo et al. 2021; Spiegel et al. 2023). Displacement exerts additional pressure on food security for both migrant and host communities. Many displaced farmers lose access to their land, complicating their ability to resume agricultural activities, resulting in heightened food insecurity (Jacobs & Almeida 2020; Tirivangasi et al. 2023). In Mozambique’s Nampula province, for instance, the influx of migrants from areas affected by conflict and climate disasters has driven up food prices, straining limited local resources and creating additional financial challenges for residents struggling with food access (Jacobs & Almeida 2020; Rossi et al. 2024). In Zimbabwe, displaced individuals in informal urban settlements face limited land and purchasing power access, exacerbating their vulnerability to food shortages (Muromo & Mashingaidze 2024). Communal farming practices, which are a cornerstone of rural food production, are often disrupted by forced relocations, fragmenting agricultural systems and reducing output, further amplifying food scarcity (Jacobs & Almeida 2020; Bratton & Masunungure 2007; Moyo 2000; Mucherera & Spiegel 2022; Muromo & Mashingaidze 2024; Takaindisa 2021). The compounded effects of displacement, agricultural losses and destabilised food systems contribute to heightened malnutrition, particularly affecting vulnerable groups such as children, pregnant women and the elderly (Mugabe et al. 2021; Ndlovu et al. 2024b; Nzima & Maviza 2023). In Zimbabwe, chronic malnutrition (stunting) impacts around 38% of children under five, with climate change and displacement further worsening these conditions (Chatiza 2019; Mutizwa 2023). Similarly, acute malnutrition remains high in Mozambique’s cyclone-affected regions, especially in temporary shelters with limited access to nutritious food (Mugambiwa & Makhubele 2021; Rossi et al. 2024; Spiegel et al. 2023). A key divergence in the literature concerns the underlying factors that shape these outcomes. While both nations face similar climatic shocks, Zimbabwe’s food security crisis is frequently analysed in the context of profound economic instability, which critically hampers public investment in climate-resilient agriculture and social safety nets (Mkodzongi 2023). In contrast, Mozambique’s challenges are often framed more around the sheer frequency and intensity of coastal hazards overwhelming disaster management systems, despite having more established policy frameworks (Chatiza 2019; Mutizwa 2023; Ndlovu et al. 2024a; Tirivangasi et al. 2023). This contextual difference is crucial for understanding the distinct policy challenges each country faces. Coordinated efforts to restore agricultural systems, improve resilience and secure sustainable food sources are crucial to preventing rising hunger and nutrition risks amid climate-induced displacements.

### Climate-induced displacement and water (in)security

Water security is equally undermined by the cycle of displacement triggered by climate disasters. Mozambique and Zimbabwe grapple with severe water availability challenges intensified by climate-induced disasters like cyclones, floods and droughts, which disrupt access to essential resources and trigger large-scale displacement (Chatiza 2019; Ekblom 2012; Mugabe et al. 2021; Spiegel et al. 2023; Trummer et al. 2023). These disasters destroy infrastructure and housing and escalate water security risks, forcing displaced populations to confront compounding challenges such as limited access to water, food and healthcare and increased risk of conflict in both countries (Nhamo et al. 2021; Spiegel et al. 2023). The recurrent displacement crises underscore the vulnerability of these regions’ infrastructure and social services, worsening conditions for internally displaced persons (IDPs) and migrants (Jacobs & Almeida 2020; Takaindisa 2021). Mozambique’s Zambezi and Limpopo River Basins, areas particularly vulnerable to climate change, have seen significant displacement because of recurring natural hazards. Cyclones Idai and Kenneth in 2019 were particularly catastrophic, displacing over 200 000 people along Mozambique’s coastline and devastating infrastructure across Sofala, Zambézia and Manica provinces (Mugabe et al. 2021; Nhamo et al. 2021; Tirivangasi et al. 2023). The eastern regions of Zimbabwe, such as Chimanimani and Chipinge, experienced similar destruction given their geographic proximity to Mozambique, leading to widespread displacement and infrastructure collapse (Nhamo et al. 2021). This cyclical displacement perpetuates vulnerability, as IDPs frequently settle in areas also prone to environmental risks. The destructive impact on infrastructure is recurrent. Cyclone Gombe (2022) ravaged Mozambique’s Zambézia and Nampula provinces, damaging 69 health facilities, obliterating more than 80% of homes in the Maratane settlement, which hosts approximately 9000 IDPs (Jacobs & Almeida 2020; Mugabe et al. 2021; Ndlovu et al. 2024b). In Zimbabwe, cyclone-induced floods have repeatedly damaged infrastructure, leaving IDPs in precarious makeshift shelters and vulnerable to future climate events (Muromo & Mashingaidze 2024; Ndlovu et al. 2024a; Nhamo et al. 2021; Tirivangasi et al. 2023). Many of these flood-related disasters in recent years have occurred in Tokwe Mukosi, Masvingo, Tsholotsho and Kanyemba Mbire District, leaving communities reliant on precarious, makeshift water sources (Mugambiwa & Makhubele 2021). These flood-induced disasters have been reported to have far-reaching consequences, affecting multiple aspects of human security, infrastructure and the environment. The consequences are dire: contaminated water and poor sanitation in overcrowded camps have led to deadly cholera outbreaks, as seen after Cyclone Idai in Mozambique (Mugambiwa & Makhubele 2021). Water scarcity also directly fuels conflict. In drought-stricken areas like Tsholotsho, Zimbabwe, tensions and occasional violence erupt over access to limited boreholes and wells (Chatiza 2019). Furthermore, the strain on water resources extends to livestock health, undermining a critical asset for household recovery and economic resilience in both agrarian societies (Mucherera & Spiegel 2022). The gendered dimensions of these crises are consistently emphasised. Women and girls, typically responsible for water collection and household food security, bear a disproportionate burden. They face increased risks of gender-based violence during long journeys to fetch water in drought-affected Zimbabwe or while navigating insecure displacement camps in Mozambique (Diab & Scissa 2023; Maviza et al. 2024). This consensus across studies underscores the imperative for gender-sensitive humanitarian and adaptation responses.

### Compounding risks associated with food and water security

This section examines the multifaceted risks that compound the primary impacts of food and water insecurity for displaced populations. While the literature consistently documents severe health and social consequences, the analysis reveals significant divergences in how these risks manifest and are addressed in Mozambique and Zimbabwe.

### Nutrition risks

Climate-induced displacement creates a cascade of risks that directly threaten nutrition and public health. A broad consensus in the literature confirms that displacement disrupts dietary intake, access to markets and traditional food systems, forcing vulnerable populations into harmful coping strategies (Cau & Agadjanian n.d.; Jagoe et al. 2023; Mazenda & Mushayanyama 2024). The destruction of agricultural livelihoods, a common consequence of floods, droughts and cyclones, eliminates both a primary food source and income, leading directly to heightened risks of malnutrition and hunger (Ndlovu et al. 2024a; Spiegel et al. 2023). The conditions within displacement settings act as critical risk multipliers. Overcrowded camps with inadequate sanitation and limited clean water create environments ripe for disease outbreaks, which further deplete nutritional status (Mugabe et al. 2021; Trummer et al. 2023). This synergy between infection and malnutrition is particularly devastating for children. In Mozambique, for instance, studies directly link nutritional deficits to impaired child development and high childhood mortality (54.0 per 1000 live births) in post-disaster contexts (Mugabe et al. 2021). Similarly, in Zimbabwe, chronic child stunting remains a pervasive concern exacerbated by recurrent climate shocks (Mutizwa 2023). A primary health risk stems from the contamination of water sources following floods or the collapse of sanitation infrastructure. This has led to deadly outbreaks of cholera and typhoid in both countries, as displaced populations are forced to rely on polluted water (Chatiza 2019; Nhamo et al. 2021). The pressure on overburdened health systems, often lacking infrastructure and personnel in hosting areas, leaves outbreaks poorly managed and amplifies the overall human cost of displacement (Jacobs & Almeida 2020). However, a key divergence emerges in the literature regarding the underlying drivers of these health crises. In Mozambique, the health narrative is dominated by acute, disaster-driven epidemics following cyclones and floods (e.g. cholera post-Idai). In Zimbabwe, while flooding is a concern, the health discourse is more frequently tied to chronic water scarcity from prolonged droughts, which leads to poor hygiene practices and water-related diseases even without a single catastrophic event. This distinction points to different priorities: outbreak containment and rapid water-system restoration in Mozambique versus long-term water supply solutions and hygiene promotion in Zimbabwe.

### Comparative analysis

A comparative examination of Mozambique’s and Zimbabwe’s responses to climate-induced displacement highlights key differences in addressing social conflicts, human security and policy measures. Although both countries contend with the compounded impacts of climate events on displaced populations, Mozambique’s approach to conflict management and disaster preparedness contrasts with Zimbabwe’s more limited focus on adaptation and resilience-building (Jacobs & Almeida 2020; Mkodzongi 2023). The differing risk profiles in the two countries are mirrored in their policy responses, a point of significant discussion in the literature. Mozambique has developed a more institutionalised DRR framework with early warning systems (EWSs) and cyclone preparedness, partly necessitated by its high frequency of coastal hazards (Jacobs & Almeida 2020). However, this system is often overwhelmed by complex emergencies where disaster-response intersects with conflict, revealing limitations in operational capacity and coordination.

Zimbabwe’s approach, while articulated in policies like the National Climate Policy, is frequently described as weaker in implementation and underfunded, leading to a cycle of reactive humanitarian response rather than proactive resilience-building (Mkodzongi 2023). The country’s profound economic challenges severely constrain its ability to invest in the climate-resilient infrastructure and social protection systems needed to mitigate displacement risks. This comparative analysis underscores that context is paramount. The compounded risks faced by displaced populations are not uniform. In Mozambique, the extreme convergence of climate disaster and armed conflict creates a unique crisis of protection and survival. In Zimbabwe, the interplay of climate shocks with economic fragility and governance challenges fuels a different crisis – one of deteriorating resilience and socially destabilising competition. Recognising this divergence is essential for formulating effective, context-specific recommendations that move beyond generic solutions to address the root causes of compounded insecurity in each setting.

### Policies and response mechanisms (adaptation measures)

Analysing Mozambique’s and Zimbabwe’s responses to climate-induced displacement reveals distinct approaches to managing human security risks and implementing adaptation strategies. Mozambique has made strides in disaster management, particularly in collaboration with international organisations (Jacobs & Almeida 2020). Key measures include establishing EWSs and constructing cyclone-resistant shelters in vulnerable areas. These interventions align with broader efforts to strengthen communities’ abilities to ‘anticipate, cope with, adapt, and recover’ from climate-related shocks. However, the literature consistently highlights that long-term resilience in Mozambique will require sustained investment in climate-resilient infrastructure and support for secure, sustainable livelihoods for displaced populations (Nhamo et al. 2021). To mitigate climate risks and prevent future disasters, the government of Mozambique, through its National Institute for Disaster Management (INGC), has implemented a strategic approach: relocating people from high-risk areas to safer regions to minimise the impact of potential disasters. While this measure aims to reduce future losses, several studies note that resettlement policies generate complex challenges for those involved, whether it is the displaced individuals or the original inhabitants of the resettlement areas (Jacobs & Almeida 2020; Maviza et al. 2024; Nzima & Maviza 2023). The literature reflects a clear divergence of views: some authors interpret relocation as a necessary protective measure, whereas others highlight its social, economic and land-rights implications. Recognising these differing perspectives is crucial, as it strengthens the understanding of why certain policies remain contested and why long-term solutions must be attentive to issues of equity and local legitimacy (Chatiza 2019; Maviza et al. 2024; Ndlovu et al. 2024b; Rossi et al. 2024; Spiegel et al. 2023). Evidence reviewed for this study points to inconsistencies within Mozambique’s legal framework, including the gradual weakening of customary land protections, the absence of coherent regulations governing expropriation and limited attention to sustainable, long-term resettlement planning (Jacobs & Almeida 2020; Maviza et al. 2024). Zimbabwe’s response to drought-induced displacement shows a different pattern, shaped by persistent political and economic constraints that limit the state’s capacity to address structural drivers of vulnerability. The literature highlights the need for improvements in water resource management, drought-resistant agricultural practices and stronger social protection systems if the country is to reduce recurrent displacement linked to climate variability (Mugabe et al. 2021; Tirivangasi et al. 2023). Zimbabwe’s preparedness is further weakened by inadequate emergency management systems, insufficient budgets, lack of political will and insufficient community education campaigns (Mutizwa 2023). Research suggests that the Zimbabwean government remains caught in the disaster-response cycle instead of adopting a proactive approach to prevention (Mavhura 2020). This mirrors broader regional patterns in Southern Africa, where governments have not sufficiently prioritised drought-resilience building over reactive responses; instead, they are caught in cyclical responses to recurring droughts (Kamara et al. 2018). As a result, adaptation policies in Zimbabwe remain underfunded, leaving displaced populations vulnerable to ongoing environmental shocks and resource scarcity and insecurities (Mucherera & Spiegel 2022). Compounding these challenges are weak or non-existent legal safeguards for communities affected by large-scale environmental interventions. Studies demonstrate that dam construction projects, for example, have disrupted water flows, altered disease ecologies and displaced households while fracturing local social relations and livelihoods (Muromo & Mashingaidze 2024). Reviewing institutional responses to cyclones further reveals that disaster risk management systems in both countries involve multiple agencies but remain hampered by limited technical, financial and logistical capacities, resulting in approaches that are more reactive than preventive (Spiegel et al. 2023; Trummer et al. 2023). In summary, while Mozambique and Zimbabwe confront climate-induced displacement, their approaches reflect different priorities and institutional realities. Mozambique’s focus on immediate disaster management underscores a short-term but structured strategy, albeit one complicated by contested land governance frameworks. In contrast, Zimbabwe’s challenges call for strengthened long-term adaptation policies that address the structural drivers of displacement and enhance community resilience. The contrast between these strategies reinforces the importance of context-specific responses to climate-induced displacement, tailored to each country’s unique challenges and institutional capacities.

## Conclusion

This study demonstrates that climate-induced displacements in Mozambique and Zimbabwe are not isolated occurrences but are part of a broader pattern of environmental degradation, economic instability and social insecurity. By reviewing 32 articles and reports, the study identifies the primary drivers and impacts of displacement caused by recurrent droughts, cyclones and floods. The findings confirm that extreme weather events, such as Cyclone Idai (2019), Cyclone Gombe (2022) and Cyclone Freddy (2023), illustrate how extreme weather intensifies food and water insecurity, undermines livelihoods and heightens social tensions, particularly within communities already experiencing deep structural vulnerabilities. These findings directly address the research question by outlining the extent of climate-induced displacement and the multi-dimensional human security challenges it creates, including constrained access to food, water, land and stable livelihood. The analysis reveals that the effects of climate change undermine human security (food and water security), which is central to long-term national development and growth (Mutizwa 2023; Rossi et al. 2024). Recurrent climate shocks, such as cyclones, floods and droughts, have compounded the vulnerabilities of fragile communities in both countries, producing displacement alongside health risks, resource-based conflicts and socio-economic instability (Mavhura 2020; Trummer et al. 2023). Future climate projections for Southern Africa indicate that the frequency and severity of such events will intensify (Mutizwa 2023; Ndlovu et al. 2024a; Maviza et al. 2024), yet Zimbabwe and Mozambique still face significant limitations in preparedness and adaptive capacity because of governance weaknesses, inadequate infrastructure and constrained political and economic environments (Chatiza 2019). A central contribution of this study is the recognition that Mozambique and Zimbabwe do not approach climate-induced displacement in the same way, and these differences significantly shape outcomes. Mozambique’s response has largely centred on immediate disaster management through EWSs, evacuation protocols and resettlement initiatives, reflecting a short-term, event-driven adaptation strategy. However, its resettlement programmes remain contested because of gaps in land governance and inconsistencies within legal frameworks (Jacobs & Almeida 2020; Maviza et al. 2024). In contrast, Zimbabwe’s response capacity is constrained by chronic economic and political challenges, leaving the country caught in a reactive disaster-response cycle and struggling to address structural drivers of drought-related displacement (Mucherera & Spiegel 2022; Mugabe et al. 2021). These divergences underscore that climate-induced displacement is profoundly shaped by national context; governance, institutional capacity, economic stability and political priorities, not by climate impacts alone.

Recognising these contextual differences is critical for designing meaningful interventions. A uniform regional approach risks overlooking the unique institutional constraints and governance realities that determine how each country can respond. The findings therefore highlight the urgent need for context-specific policy measures: Mozambique requires strengthened long-term resettlement planning and land-rights protections to complement its disaster-management focus, while Zimbabwe must prioritise drought-resilience building, social safety nets and governance reforms to escape its recurring crisis-response cycle. Across both countries, improved disaster preparedness, investment in climate-resilient infrastructure and expanded social protection systems are essential to safeguard human security and reduce displacement risks. Overall, this study concludes that decisive, context-sensitive action is imperative. Without targeted interventions that reflect the divergent pathways and constraints of Mozambique and Zimbabwe, climate-induced displacement will escalate, deepening human suffering and undermining long-term development trajectories in both countries.

### Recommendations

From the above findings and conclusions, the study makes the following recommendations to ensure that Mozambique and Zimbabwe can better manage climate-related displacements while safeguarding human security and ensuring long-term resilience for affected populations:

- National Adaptation Plans (NAPs) in Mozambique and Zimbabwe must integrate targeted measures to address climate change-induced displacement. Key provisions should include the development of climate-resilient infrastructure, strengthening social protection systems to support displaced populations and incorporating DRR strategies in the NAPs. Furthermore, NAPs must prioritise community-centred approaches that systematically incorporate local knowledge and address specific community needs.
- Governments in Zimbabwe and Mozambique should strengthen EWSs to mitigate displacement risks triggered by extreme weather events such as cyclones, floods and droughts. Governments should allocate resources to advance climate monitoring technology, enhance the accuracy and timeliness of data collection and analysis and ensure the localisation of EWS for effective dissemination of information to vulnerable communities through accessible channels. Regular assessments of EWS are essential to ensure adaptability to evolving climate patterns and emerging risks.
- There is a need for governments to establish and enhance comprehensive disaster preparedness and response frameworks. This requires equipping and training local response teams, strategically pre-positioning essential supplies in high-risk areas and implementing well-designed evacuation plans for vulnerable communities. Integrating these measures into broader developmental strategies will enhance resilience, reduce the adverse impacts of climate-induced disasters and promote sustainable adaptation in the face of climate challenges.
- Enhancing community participation and engagement by involving local communities, traditional leaders and vulnerable groups in planning and implementing resettlement programmes to ensure culturally sensitive and equitable outcomes
- Advocate for increased access to climate finance and international funding support adaptation measures, resettlement projects and infrastructure development.
